# Hybrid topoisomerase I and HDAC inhibitors as dual action anticancer agents

**DOI:** 10.1371/journal.pone.0205018

**Published:** 2018-10-09

**Authors:** Raffaella Cincinelli, Loana Musso, Roberto Artali, Mario B. Guglielmi, Ilaria La Porta, Carmela Melito, Fabiana Colelli, Francesco Cardile, Giacomo Signorino, Alessandra Fucci, Martina Frusciante, Claudio Pisano, Sabrina Dallavalle

**Affiliations:** 1 Department of Food, Environmental and Nutritional Sciences, Università degli Studi di Milano, Milano, Italy; 2 Scientia Advice, Desio, Monza and Brianza, Italy; 3 Biogem, Research Institute, Ariano Irpino, Avellino, Italy; Florida International University, UNITED STATES

## Abstract

Recent studies have shown that HDAC inhibitors act synergistically with camptothecin derivatives in combination therapies. To exploit this synergy, new hybrid molecules targeting simultaneously topoisomerase I and HDAC were designed. In particular, a selected multivalent agent containing a camptothecin and a SAHA-like template showed a broad spectrum of antiproliferative activity, with IC_50_ values in the nanomolar range. Preliminary *in vivo* results indicated a strong antitumor activity on human mesothelioma primary cell line MM473 orthotopically xenografted in CD-1 nude mice and very high tolerability.

## Introduction

Over the last two decades, drug discovery has been primarily focused on the development of single-target drugs with high potency and selectivity. However, single target agents can perform excellently during the treatment of diseases with linear pathways. Multi-factorial diseases with complex signalling networks, such as cancer, are robust and can be efficiently eradicated only by the simultaneous attack of multiple targets.

A well-established strategy for anticancer therapy is the rational design of drug combinations aimed at achieving synergistic effects and overcoming drug resistance. However, the combination of agents that exhibit synergistic interaction in cell culture can be less effective than expected. This is mainly due to differential pharmacokinetic behaviour of each drug and to the difficulty to afford optimal concentrations for the required time [1]. Other shortcomings of drug combinations are unpredictable drug-drug interactions and possible enhancement of adverse effects. To overcome these limitations, an increase of interest has been made recently in hybrid bifunctional agents designed to inhibit simultaneously multiple cellular targets relevant to tumour growth/survival [2–5]. The benefits of drugs with multiple targets could be (a) improvement of the efficacy by exploiting synergistic interactions; (b) enhancement of the selectivity resulting in a better tolerability; (c) modulation of drug resistance. Additionally, multivalent molecules are expected to provide pharmacokinetic and pharmacodynamic advantages over the separate administration of the two drug components [4, 5].

**Fig 1:**
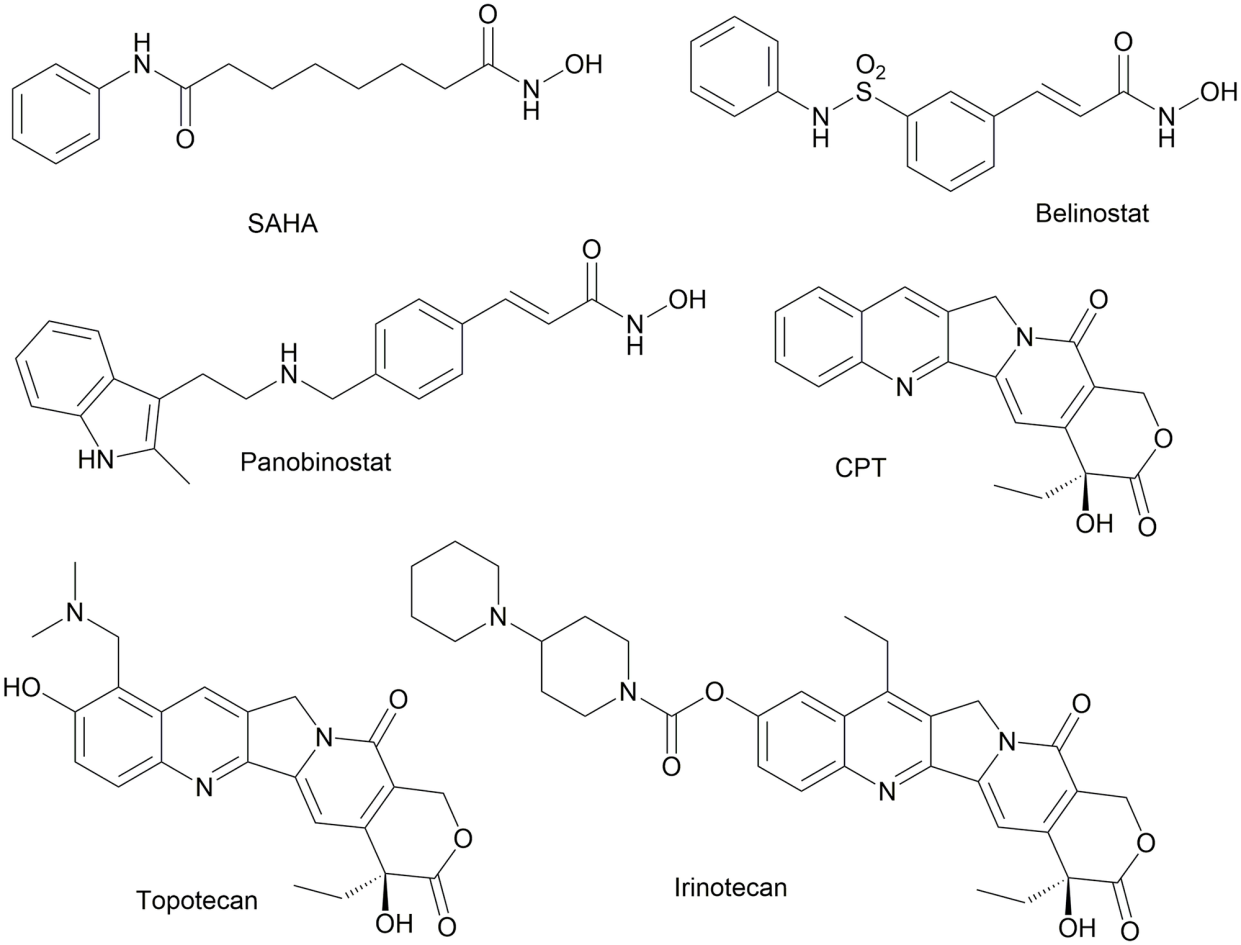
Structures of representative HDAC and topoisomerase I inhibitors.

One attractive pathway for tumour growth inhibition is the protein acetylation state modulation by histone deacetylases (HDACs). To date, 18 human HDACs have been identified and grouped into four classes on the basis of their homology with yeast proteins [6]. Class I HDACs (HDAC1, 2, 3, and 8) share high homology with the yeast transcriptional regulator RPD3; class II HDACs are closely related to HDAC1 (HDAC4, 5, 6, 7, 9, and 10); class III HDACs, also called sirtuins, are homologous with Sir2 (SIRT1, 2, 3, 4, 5, 6, and 7), and class IV HDAC (HDAC11) is homologous with class I and II enzymes. HDAC inhibitors have already surpassed the clinical trial stage, culminating with the approval of Vorinostat, originally known as SAHA (suberoylanilide hydroxamic acid), romidepsin (FK228), panobinostat (LBH-589), and belinostat (formerly PXD-101) [6, 7] (Fig 1).

Another class of therapeutic agents acting at a nuclear level are topoisomerase I inhibitors [8, 9]. Topoisomerases relieve the torsional stress associated with the separation of DNA strands during transcription and DNA replication. Eukaryotic Topoisomerase I (Top1) is a Type IB enzyme that nicks and rejoins only one strand of duplex DNA, and it is critically important for maintaining genome integrity [10]. Many small molecule inhibitors of Top1 have proven clinically effective, the most potent being the camptothecin (CPT) derivatives [11, 12] (Fig 1).

Two semisynthetic camptothecins have been approved by US FDA for cancer chemotherapy: Irinotecan (CPT-11) and Topotecan. Belotecan (Camtobell) has been approved in South Korea for treatment of NSCLC (Fig 1). Other derivatives are in different stages of clinical development, such as Lipotecan [13] and BAY 56–3722 [14], together with a number of macromolecular delivery agents and supramolecular assemblies [15].

The cancer-promoting mechanism of HDAC involves transcriptional silencing of tumor suppressors via deacetylation of nucleosomes containing tumor suppressor genes. HDAC inhibition has been reported to affect cancer cells mainly by a global relaxation of chromatin structure, which contributes to a recovered cell response to chemotherapeutic agents targeting DNA [16]. In this respect, there is a lot of evidence showing that co-treatment with HDAC inhibitors and camptothecin derivatives (such as Topotecan, Irinotecan) synergistically block cell proliferation [17–24]. We decided to exploit this synergistic effects by designing hybrid inhibitors which could, in principle, interact with both targets. In particular, we chose to incorporate into a prototypical HDACi pharmacophore a properly substituted CPT. Classical medicinal chemistry approaches coupled with computational analysis and biological evaluation led to the definition of key molecular insights for dual inhibition.

## Materials and methods

### Chemistry

#### General information

All reagents and solvents were reagent grade or were purified by standard methods before use. Melting points were determined in open capillaries and are uncorrected. NMR spectra were recorded in CDCl_3_ (where not otherwise stated) at 300 MHz. Chemical shifts (δ values) and coupling constants (J values) are given in ppm and Hz, respectively. Solvents were routinely distilled prior to use; anhydrous tetrahydrofuran (THF) and ether (Et_2_O) were obtained by distillation from sodium-benzophenone ketyl; dry methylenechloride was obtained by distillation from phosphorus pentoxide. All reactions requiring anhydrous conditions were performed under a positive nitrogen flow, and all glassware were oven dried and/or flame dried. Isolation and purification of the compounds were performed by flash column chromatography on silica gel 60 (230–400 mesh). Analytical thin-layer chromatography (TLC) was conducted on Fluka TLC plates (silica gel 60 F254, aluminum foil). Analyses indicated by the symbols of the elements or functions were within ±0.4% of the theoretical values. Compounds **2a** [25], **1a**, **2b**, **2c**, [3], **5** [26], 7-formylcamptothecin and 10-hydroxy-7-formylcamptothecin (**1b**) [27] were prepared according to literature procedures.

#### Synthesis of compounds 3a-d, 10

**7-(camptothecin-7-ylmethyleneaminooxy)-heptanoic acid (2c)**. To a solution of **1a** (200 mg, 0.47 mmol) in acetic acid (15 ml) with 7-aminooxyheptanoic acid hydrobromide (250 mg, 1.04 mmol) was added and the mixture heated 3 h at 80 °C. The solvent was evaporated and the residue was purified by flash chromatography with CH_2_Cl_2_:CH_3_OH 93:7 to give 171 mg (70%) of compound **2c**. mp:124–125 °C. ^1^H-NMR (300 MHz, CDCl_3_)δ: 10.50 (1H, brs); 8.84 (1H, s); 8.09 (1H, d, J = 8.5 Hz); 8.01 (1H, d, J = 8.5 Hz); 7.98 (1H, s); 7.69 (1H, dd, J = 8.5, 8.5 Hz); 7.58 (1H, s); 7.53 (1H, dd, J = 8.5, 8.5 Hz); 5.70–5.60 (1H, m); 5.28–5.15 (3H, m); 4.34 (2H, t, J = 6.3 Hz); 2.32 (2H, t, J = 6.9 Hz); 1.91–1.77 (4H, m); 1.71–1.57 (2H, m); 1.51–1.35 (4H, m); 0.89 (3H, t, J = 7.1 Hz). Anal. calcd for C_28_H_29_N_3_O_7_: C,64.73; H, 5.63; N, 8.09; Found: C, 64.65; H, 5.61; N, 8.10.

**Camptothecin-7-yl(methyleneaminooxy)-acetic acid hydroxyamide (3a)**. To a solution of **2a** (50 mg, 0.11 mmol) in dry DMF (1 mL), DCC (35 mg, 0.17 mmol), and N-hydroxysuccinimide (20 mg, 0.17 mmol) were added under nitrogen atmosphere. The mixture was stirred 48 h at rt, then DCC (12 mg 0.06 mmol) and N-hydroxysuccinimide (7 mg, 0.06 mmol) were added. The reaction was stirred 24 h at rt, then hydroxylamine hydrochloride (23 mg, 0.33 mmol) and TEA (44 μl, 0.33 mmol) were added and stirring was continued for 24 h at room temperature. The solvent was evaporated and the crude was purified by flash chromatography with CH_2_Cl_2_:CH_3_OH from 195:5 to 190:10 to give 5 mg of product (10%). mp: 169 °C. ^1^H-NMR (300 MHz, DMSO-*d*_*6*_) δ: 10.82 (1H, brs); 9.37 (1H, s); 9.00 (1H, brs); 8.62 (1H, d, J = 8.5 Hz); 8.24 (1H, d, J = 8.5 Hz); 7.94 (1H, dd, J = 8.5, 8.5 Hz); 7.80 (1H, dd, J = 8.5, 8.5 Hz); 7.37 (1H, s); 6.55 (1H, s); 5.44 (2H, s); 5.34 (2H, s); 4.78 (2H, s); 1.99–1.77 (2H, m); 0.89 (3H, t, J = 7.0 Hz). Anal. calcd for C_23_H_20_N_4_O_7_: C,59.48; H, 3.34; N, 12.06; Found: C, 59.63; H, 3.35; N, 12.04.

**6-(camptothecin-7-ylmethyleneaminooxy)-hexanoic acid hydroxyamide (3b)**. To a solution of **2b** (350 mg, 0.69 mmol) in dry DMF (6 ml), WSC (172 mg, 0.9 mmol) and HOBt (112 mg, 0.83 mmol) were added under nitrogen atmosphere. The mixture was stirred 3 h at rt. cooled to 0 °C, and hydroxylamine hydrochloride (240 mg, 3.45 mmol) and TEA (0.48 ml, 3.45 mmol) were added. The reaction was stirred 1 h at room temperature. The solvent was evaporated and the residue was purified by flash chromatography with CH_2_Cl_2_:CH_3_OH 91: 9 to give 220 mg of product (61%). mp: 158–159°C. ^1^H-NMR (300 MHz, DMSO-*d*_*6*_) δ: 10.36 (1H, s); 9.33 (1H, s); 8.68 (1H, s); 8.60 (1H, d, J = 8.3 Hz); 8.21 (1H, d, J = 8.3 Hz); 7.91 (1H, dd, J = 8.3, 8.3 Hz); 7.76 (1H, dd, J = 8.3, 8.3 Hz); 7.36 (1H, s); 6.55 (1H, s); 5.44 (1H, s); 5.32 (1H, s); 4.35 (2H, t, J = 6.5 Hz); 2.00 (2H, t, J = 7.3 Hz); 1.94–1.75 (4H, m); 1.65–1.52 (2H, m); 1.51–1.38 (2H, m); 0.89 (3H, t, J = 7.0 Hz). Anal. calcd for C_27_H_28_N_4_O_7_: C,62.30; H, 5.42; N, 10.76; Found: C, 62.24; H, 5.43; N, 10.74.

**7-(camptothecin-7-ylmethyleneaminooxy)-heptanoic acid hydroxyamide (3c)**. To a solution of **2c** (150 mg, 0.29 mmol) in dry DMF (2.5 ml), WSC (72 mg, 0.38 mmol) and HOBt (46 mg, 0.35 mmol) were added under nitrogen atmosphere. The mixture was stirred overnight at room temperature. The reaction was cooled to 0 °C and hydroxylamine hydrochloride (101 mg, 1.45 mmol), TEA (0.2 mL, 1.45 mmol) were added. The mixture was stirred 1 h at rt. The solvent was evaporated and the residue purified by flash chromatography with CH_2_Cl_2_:CH_3_OH 91:9 to give 70 mg of product (45%). mp: 164–165°C. ^1^H-NMR (300 MHz, DMSO-*d*_*6*_) δ: 10.33 (1H, s); 9.31 (1H, s); 8.65 (1H, s); 8.58 (1H, d, J = 8.5 Hz); 8.21 (1H, d, J = 8.5 Hz); 7.90 (1H, dd, J = 8.5, 8.5 Hz); 7.75 (1H, dd, J = 8.5, 8.5 Hz); 7.35 (1H, s); 6.53 (1H, s); 5.43 (2H, s); 5.30 (2H, s); 4.41–4.27 (2H, m); 2.56–2.38 (2H, m); 2.03–1.68 (4H, m); 1.64–1.23 (6H, m); 0.90 (3H, t, J = 7.0 Hz). Anal. calcd for C_28_H_30_N_4_O_7_: C,62.91; H, 5.66; N, 10.48; Found: C, 62.98; H, 5.64; N, 10.50.

**6-(10-hydroxycamptothecin-7-ylmethyleneaminooxy)-hexanoic acid hydroxyamide (3d)**. To a solution of **2d** (287 mg, 0.56 mmol) in dry DMF (20 ml), WSC (140 mg, 0.73 mmol) and HOBt (140 mg, 0.73 mmol) were added. The mixture was cooled to 0 °C and hydroxylamine hydrochloride (82 mg, 1.18 mmol) and TEA (111 μl, 1.18 mmol) were added. The reaction was stirred 8 h at room temperature, the solvent was evaporated and the residue was diluted with water (40 mL). The resulting solid was filtered to obtain 220 mg of product (73%). mp: 202–203 °C. ^1^H-NMR (300 MHz, DMSO-*d*_*6*_) δ: 10.49 (1H, brs); 10.36 (1H, s); 9.02 (1H, s); 8.66 (1H, brs); 8.06 (1H, d, J = 9.2 Hz); 7.72 (1H, d, J = 1.8 Hz); 7.47 (1H, dd, J = 1.8, 9.2 Hz); 7.26 (1H, s); 6.50 (1H, s); 5.41 (2H, s); 5.27 (2H, s); 4.33 (2H, t, J = 5.8 Hz); 2.04–1.93 (2H, m); 1.92–1.72 (4H, m); 1.66–1.52 (2H, m); 1.51–1.35 (2H, m); 0.89 (3H, t, J = 7.0 Hz). Anal. calcd for C_27_H_28_N_4_O_8_: C,60.44; H, 5.26; N, 10.44; Found: C, 60.55; H, 5.27; N, 10.41.

**2-(6-aminohexyloxy)-isoindole-1,3-dione) trifluoroacetate (6)**. To a solution of compound **5** (835 mg, 2.3 mmol) in CH_2_Cl_2_ (5 mL) trifluoroacetic acid (2 mL) and was added and the mixture was stirred 40 min at room temperature. Trifluoroacetic acid and solvent were removed by evaporation under vacuo and the residue was grounded with ethyl ether, to give 810 mg (94%) of product. mp:99 °C. ^1^H-NMR (300 MHz, DMSO-*d*_*6*_) δ: 7.89–7.51 (4H, m); 4.13 (2H, t, J = 6.4 Hz); 2.86–2.72 (2H, m); 1.73–1.29 (8H, m).

**N-[6-(1,3-dioxo-1,3-dihydro-isoindol-2-yloxy)-hexyl]-2-tritylsulfanylacetamide (7)**. To a solution of **6** (300 mg, 0.80 mmol) in CH_2_Cl_2_ (3 ml), tritylsulfonylacetic acid (261 mg, 0.78 mmol), DMAP (5 mg, 0.04 mmol), TEA (167 μl, 1.2 mmol), and WSC (199 mg, 1.04 mmol) were added under nitrogen atmosphere. The mixture was stirred 1.5 h at room temperature. After dilution with dichloromethane, the mixture was washed with brine, dried and evaporated. Purification by flash chromatography with hexane:EtOAc 6:4, gave 238 mg (53%) of product. mp: 133 °C. ^1^H-NMR (300 MHz, DMSO-*d*_*6*_) δ: 7.88–7.70 (4H, m); 7.48–7.17 (15H, m); 6.06 (1H, brs); 4.20 (2H, t, J = 6.4 Hz); 3.14 (2H, s); 3.02–2.91 (2H, m); 1.85–1.68 (2H, m); 1.56–1.44 (2H, m); 1.43–1.23 (4H, m). Anal. calcd for C_35_H_34_N_2_O_4_S: C,72.64; H, 5.92; N, 4.84; Found: C, 72.48; H, 5.93; N, 4.85.

***N*-(6-Aminooxyhexyl)-2-tritylsulfanyl-acetamide (8)**. To a solution of **7** (94 mg, 0.16 mmol) in methanol (2 ml), NH_2_NH_2_.H_2_O (25 μL, 0.48 mmol) was added and the solution was heated to reflux for 1h. After evaporation of the solvent, the residue was diluted with ethyl acetate. The solid was filtered and the solvent evaporated under reduced pressure. Purification by flash chromatography with hexane:EtOAc 2:1 gave 60 mg of an oil (83%). ^1^H-NMR (CDCl_3_) δ: 7.48–7.12 (15H, m); 6.01 (1H, brs); 5.35 (1H, brs); 3.65 (2H, t, J = 6.7 Hz); 3.14 (2H, s); 2.99–2.89 (2H, m); 1.44–1.08 (8H, m). Anal. calcd for C_27_H_32_N_2_O_2_S: C,72.29; H, 7.19; N, 6.24; Found: C, 72.09; H, 7.20; N, 6.23.

**N-[6-(camptothecin-7-ylmethyleneaminooxy)-hexyl]-2-tritylsulfanylacetamide (9)**. To a solution of 7-formylcamptothecin (25 mg, 0.07 mmol) in ethanol (2 mL) *N*-(6-aminooxyhexyl)-2-tritylsulfanylacetamide (**8**) (25 mg, 0.11 mmol) was added and the mixture was heated 5h to reflux. The solvent was evaporated and the crude was purified by chromatography with CH_2_Cl_2_:CH_3_OH 98:2 to obtain 51 mg of a yellow solid (91%). mp: 108 °C. ^1^H-NMR (300 MHz, DMSO-*d*_*6*_) δ: 9.32 (1H, s); 8.59 (1H, d, J = 8.5 Hz); 8.22 (1H, dd, J = 8.5, 8.5 Hz); 7.90–7.69 (2H, m); 7.40–7.15 (16H, m); 6.56 (1H, s); 5.42 (2H, s); 5.30 (2H, s); 4.34 (2H, t, J = 6.4 Hz); 3.02–2.89 (2H, m); 2.75 (2H, s); 1.94–1.69 (4H, m); 1.49–1.07 (6H, m); 0.89 (3H, t, J = 7.0 Hz). Anal. calcd for C_48_H_46_N_4_O_6_S: C,71.44; H, 5.75; N, 6.94; Found: C, 71.38; H, 5.73; N, 6.95.

**N-[camptothecin-7-yl-(methyleneaminooxy)-hexyl]-2-mercaptoacetamide (10)**. To a solution of **9** (63 mg, 0.08 mmol) in CH_2_Cl_2_ (2.6 mL) trifluoroacetic acid (78 μl, 1.02 mmol) and triethylsilane (30 μl, 0.19 mmol) were added. The solution was stirred 2.5 h at room temperature, the solvent was evaporated and the residue was purified by flash chromatography with CH_2_Cl_2_:CH_3_OH to give 36 mg of compound **10** (80%). mp:120 °C. ^1^H-NMR (300 MHz, DMSO-*d*_*6*_) δ: 9.32 (1H, s); 8.60 (1H, d, J = 8.5 Hz); 8.20 (1H, d, J = 8.1 Hz); 7.96 (1H, t, J = 5.5 Hz); 7.92–7.85 (1H, m); 7.79–7.70 (1H, m); 7.33 (1H, s); 6.53 (1H, s); 5.47–5.24 (4H, m); 4.34 (2H, t, J = 6.4 Hz); 3.10–2.99 (4H, m); 1.95–1.68 (4H, m); 1.51–1.29 (6H, m); 0.87 (3H, t, J = 7.3 Hz). Anal. calcd for C_29_H_32_N_4_O_6_S: C,61.69; H, 5.71; N, 9.92; Found: C, 61.75; H, 5.72; N, 9.94.

### Cell lines

Human primary epithelioid MM288, MM317, MM404, MM473, MM473-Luc; biphasic MM487 and MM491; sarcomatoid MM432 and MM472 mesothelioma cell lines (all from our cell bank collection).

Human K562 erythromyeloblastoid leukemia (ATCC # CCL-243), ARH-77 plasma leukemia (ATCC # CRL-1621), NB4 acute promyelocytic leukemia (ATCC # ACC-207), THP1 and MV4-11 monocytic leukemia (ATCC # TIB-202, CRL-9591), Jurkat acute T-cell leukemia (ECACC # 90112119), U937 histiocytic lymphoma (ATCC # CRL-1593), RAJI, DG-75, and RAMOS Burkitt lymphoma (DSMZ # ACC-319, ACC-83, ACC-603); MAVER, MINO, REC-1 (DSMZ # ACC-717, ACC-687, ACC-584), JECO-1, Z-138 (ATCC # CRL-3006, CRL-3001) mantle cell lymphoma; KM-H2 and L-428 Hodgkin lymphoma (DSMZ # ACC-8, ACC-197), OCI-LY3 and U-2932 diffuse large B-cell lymphoma (DSMZ # ACC-731, ACC-633), RPMI-8226 and NCI-H929 multiple myeloma (ATCC # CCL-155, CRL-9068).

Human NCI-H460 NSCLC (ATCC # HTB-177), Capan-1 pancreatic carcinoma (ATCC # HTB-79), A431 epidermoid carcinoma (ATCC # CRL-1555), HeLa cervix carcinoma (ATCC # CCL-2), A2780 and A2780-Dx multidrug-resistant ovarian carcinoma (ECACC # 93112519, 93112520), HT29 colorectal carcinoma (ECACC # 91072201), HepG2 hepatocellular carcinoma (ATCC # HB-8065), DU145 prostate carcinoma (ATCC # HTB-81).

### Cell cultures

All primary mesothelioma cell lines (MM288, MM317, MM404, MM473, MM487, MM491, MM432, MM472) were cultured in F-10 Nutrient Mixture medium supplemented with 10% Foetal Bovine Serum (FBS), 2 mM L-glutamine and gentamicin sulfate. MM473-Luc cells were cultured in Ham’s F-10 Nutrient Mixture supplemented with 10% FBS, 2 mM L-glutamine, and G418 antibiotic. A2780, A2780-Dx, ARH-77, DG-75, Jurkat, KM-H2, HeLa, L-428, NB4, NCI-H60, U937, RAJI, REC-1, RPMI-8226, and U-2932 cells were cultured in RPMI-1640 medium supplemented with 10% FBS, 2 mM L-glutamine and gentamicin sulfate. JECO-1, MAVER-2, MINO, OCI-LY3 and RAMOS cells were cultured in RPMI-1640 medium supplemented with 20% FBS, 2 mM L-glutamine and gentamicin sulfate. H929 and THP1 cells were cultured in RPMI-1640 medium supplemented with 10% FBS, 0.05 mM mercaptoethanol, L-glutamine and gentamicin sulfate. K562, MV4-11, Z-138 cells were cultured in IMDM medium supplemented with 10% FBS, L-glutamine and gentamicin sulfate. Capan-1 cells were cultured in IMDM medium supplemented with 10% FBS, 2 mM L-glutamine and gentamicin sulfate. A431, DU145, and HepG2 cells were cultured in EMEM medium supplemented with 10% FBS, 2 mM L-glutamine and gentamicin sulfate. HT29 cells were cultured in McCoy’s medium supplemented with 10% FBS, 2 mM L-glutamine and gentamicin sulfate.

Cells were maintained in an incubator at 37°C with saturated humidity and an atmosphere of 95% air and 5% CO_2_, and were sub-cultured every 2–3 days.

### Cytotoxicity assay

Human tumor cells were seeded in 96-well cell culture plastic plates, allowed to attach, and exposed to scalar concentrations of the test compounds. Cell survival was evaluated upon 72 h by the sulphorhodamine B (SRB) or MTT tetrazolium reduction assay and the IC_50_ value (drug concentration inhibiting 50% of cell growth) calculated by the ALLFIT program.

### FACS analysis

Tumor cells were seeded in 10-cm tissue culture plates, allowed to attach and incubated for 72 h with the test compounds (IC_80_ dose). After this, cells were harvested with trypsin/EDTA, rinsed with cold PBS, and fixed in 70% ice-cold ethanol at 4 °C. The day of analysis, cells were rinsed twice with cold PBS, incubated with RNase A (50 KU/ml) for 30 min. at 37°C, stained with propidium iodide (PI, 50 μg/mL) and processed on a FACScan flow cytometer (Becton Dickinson). The CellQuest software (BD Biosciences) was used to acquire data, while cell cycle analysis was performed using the ModFit software (Verity Software House).

### HDAC’s profiling

The experiments were carried out in RBC facility (http://www.reactionbiology.com/) by using in triplicate 10 concentrations of each compound starting from 10 μM. The substrate for HDAC1,2,6, and 10 was Fluorogenic peptide from p53 residues 379–382 (RHKK(Ac)AMC). The Substrate for HDAC4 was Fluorogenic HDAC Class2a Substrate (Trifluoroacetyllysine) IC_50_ values were calculated using the GraphPad Prism 4 program based on a sigmoidal dose-response equation.

### Protein analysis

NCI-H460 cells in logarithmic phase of growth were collected in Dulbecco’s 1x PBS buffer and lysed in RIPA lysis buffer (50mM Tris-HCl, 150 mM NaCl, 0,5% Na-Deoxycolate, 1% NP-40, 0.15% SDS) containing protease and phosphatase inhibitors and then centrifuged at 12000 rpm for 10 min at 4 °C. The supernatant, containing the total proteins extracts, were transferred in new vials. The protein concentration was measured by Bradford Protein Assay. Equal amounts of proteins for each sample were then resolved by SDS-PAGE and loaded on 10–12% polyacrilamide gels. The proteins were then transferred on nitrocellulose membranes. Molecular weights were estimated based upon the relative migration with molecular weight protein markers.

The non-specific binding was blocked by incubation of membranes in TBS-tween (25 mM Tris, 150 mM NaCl, 0.1% Tween 20) with 5% nonfat dry milk (BIORAD, Milan) for 60 minutes. Subsequently, the membrane was incubated overnight with primary antibodies: anti-Histone H4 (Millipore, 1:500), anti-acetyl-H4 (Santa Cruz, 1:1000) anti-p21 (Santa Cruz, 1:1000), anti-acetyl-tubulin (abcam, 1:1000), anti-α-tubulin (Sigma, 1:5000), anti β-actin (Sigma, 1:5000) and anti-Topoisomerase 1 (Santa Cruz, 1:500). After three washes with TBST blots were incubated with HRP-coniugated secondary anti-mouse and anti-rabbit (Santa Cruz, 1:5000) 1 hour at room temperature. After three washes with TBST the blots were developed with ECL substrate (GE Healthcare) and chemiluminescence detected by ChemiDoc Imaging System (Biorad). The optical density of the protein bands detected by western blotting was normalized on α -tubulin levels and analyzed by ChemiDoc software (Biorad).

### Animal model

Female 4–6 weeks old nude mice (CD1 nu/nu) were purchased from Charles River Laboratories (Calco (Lecco) Italy) and kept in polisulfone IVC cages (33.2 x 15 x 13 cm, Tecniplast) with 75 complete changes of filtered air per hour (HEPA H 14 filter); autoclaved dust-free bedding and mouse house enrichment device (Tecniplast) were used.

Animals were housed under a light-dark cycle, keeping temperature and humidity constant. Parameters of the animal rooms were assessed as follows: 22 ± 2 °C temperature, 55 ± 10% relative humidity, about 15–20 filtered air changes/hour and 12 hour circadian cycle of artificial light (7 a.m., 7 p.m.). Mice were fed with a complete irradiated pellet diet (GLP 4RF21, Mucedola) ad libitum throughout the entire duration of the experiment, and had unrestricted access to drinking water. All animal studies were conducted in accordance with Ethics approval obtained from the Italian Ministry of Health and all experiments were in accordance with the European guidelines for the care and use of laboratory animals. All procedures were performed under deep isoflurane anesthesia and at the end of experiments were sacrified by cervical dislocation.

### In vivo antitumor activity

Human epithelioid mesothelioma MM473-Luc (luciferase-expressing) cells were cultured in complete medium, checked with Mycoplasma Detection Kit (Lonza) to exclude mycoplasma contamination, counted by trypan blue dye exclusion test to assess cell viability (> 95%), re-suspended in M199 medium, and orthotopically inoculated intrapleurally (i.pl.) in CD-1 nude mice (Charles River) at a concentration of 1x10^6^/200μl M199/mouse. Compound **3d** was diluted in 10% 1:1 absolute ethanol-Cremophor solution in saline and was administered i.v. at 45 mg/kg using q4dx3w schedule. Irinotecan was administered iv at 35 mg/kg, using the schedule q4dx3. Tumor growth was evaluated weekly by using the IVIS Spectrum (PerkinElmer) in vivo technology system, through intraperitoneal injection of D-Luciferin potassium salt (100μL/10gr, PerkinElmer). After the IP D-luciferin injection, mice were anesthetized in an oxygen-rich induction chamber with 2% isofluorane, and images were captured 30 minutes after D-Luciferin injection in order to allow substrate distribution. Tumor volume was measured as average radiance that is the total flux of photons or radiance (photons/second from the surface) in each pixel, summed or integrated over the ROI area, in a square centimeter (cm^2^) of the tissue. (photons/sec/cm^2^/sr). The established maximum average radiance was 1x10^8^ photons/sec/cm^2^/sr, used as humane end-point.

### Statistical analysis

Statistical analysis was performed by using U-Test (GraphPad Prism 6). Outliers were removed by using the Rout test (Q = 10%, GraphPad Prism 6).

### Molecular modeling

The ligand molecules were obtained using a systematic conformer search followed by geometry optimisation of the lowest energy structure with MOPAC7 (PM3 Method, RMS gradient 0.0100). The DNA-Topoisomerase-I and HDAC-II models were derived from the deposited X-ray structures (Protein Data Bank entry 1T8I and 5IWG, respectively) as previously described [3].

Energy minimisations and molecular modeling calculations were performed by using the GROMACS package [28] and the AMBER-03 [29] force field, a variant of the AMBER-99 [30] potential in which charges and main-chain torsion potentials have been derived based on QM+continuum solvent calculations and each amino acid is allowed unique main-chain charges (nucleic acids have not been modified from AMBER-99).

Molecular docking experiments were performed with Autodock 4.0 [31]. We used the Lamarckian Genetic Algorithm which combines global search (Genetic Algorithm alone) to local search (Solis and Wets algorithm) [32]. Ligands and receptors were further processed using the Autodock Tool Kit (ADT) [33]. Gasteiger–Marsili charges [34] were loaded on the ligands in ADT and Cornell parameters were used for the phosphorous atoms in the DNA. Solvation parameters were added to the final structure using the Addsol utility of Autodock. Each docking consisted of an initial population of 100 randomly placed individuals, a maximum number of 200 energy evaluations, a mutation rate of 0.02, a crossover rate of 0.80, and an elitism value of 1. For the local search, the so-called pseudo-Solis and Wets algorithm was applied using a maximum of 250 iterations per local search. 250 independent docking runs were carried out for each ligand. The grid maps representing the system in the actual docking process were calculated with Autogrid. The dimensions of the grids were 80×80×80, with a spacing of 0.1 Å between the grid points and the center close to the cavity left by the ligand after its removal. The simpler inter-molecular energy function based on the Weiner force field in Autodock was used to score the docking results. Results differing by less than 1.0 Å in positional root-mean-square deviation (rmsd) were clustered together and were represented by the result with the most favorable free energy of binding. The poses were equilibrated by a 10.0 ns of partially restrained molecular dynamics simulation using the GROMACS package.

## Results and discussion

### Chemistry

We envisaged that the appropriate combination and outdistancing between the CPT scaffold and a SAHA-like portion could lead to compounds characterized by suitable size and geometrical shape for fitting both enzymes' binding pockets. In previous papers we reported that CPT- functionalization at C-7 is highly tolerable. Particularly, we synthesized 7-oxyiminomethyl derivatives, showing potent *in vitro* and *in vivo* activity [25–27, 35, 36]. For this reason, (E)-7-oxyminomethyl-CPTs were selected for conjugation to a SAHA-like pharmacophore via a suitable chain. The CPT ring system should act as a surface recognition cap group for HDAC inhibition, at the same time maintaining the Top1 inhibition activity. To investigate the optimal linker length, we designed a series of compounds containing a SAHA-like HDAC inhibitor connected to the CPT scaffold by linear alkyl chains.

**Fig 2:**
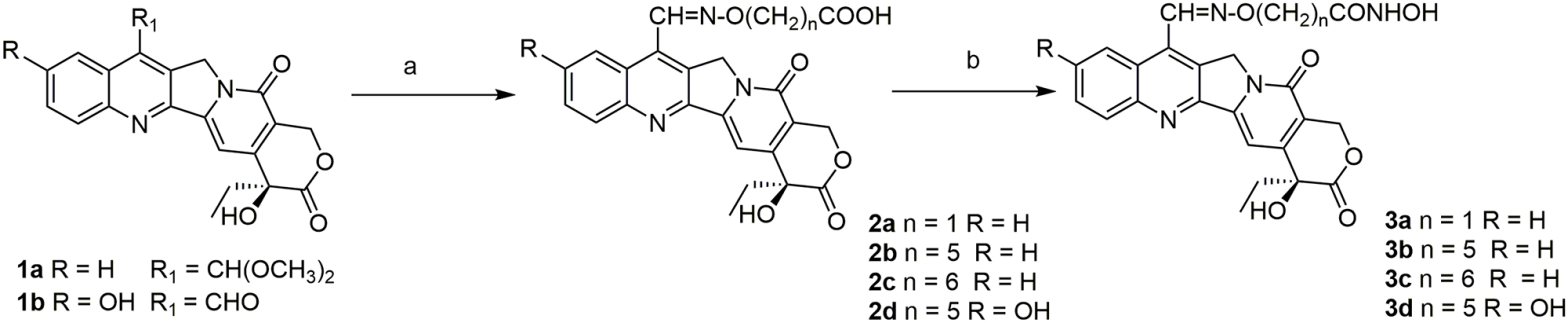
Synthesis of compounds 3a-d. Reagents and conditions: a) for **2a-c**: HCl·H_2_NO(CH_2_)_n_COOH, acetic acid, 80 °C, 3h; for **2d**: HCl·H_2_NO(CH_2_)_n_COOH, EtOH, pyridine, reflux, 4h; **2a**: 90%, **2b**: 73%, **2c**: 70%, **2d**:75%; b) for **3a**: DCC, NHS, HCl·NH_2_OH, rt, 48h, 10%; for **3b-d**, WSC, HOBt, DMF, NH_2_OH·HCl, TEA, rt, 1-8h, **3b**: 61%, **3c**: 45%, **3d**: 73%.

The reaction route to CPT-HDAC hybrid compounds **3a-c** is shown in Scheme 1. The compounds were prepared reacting the dimethylacetal of 7-formylcamptothecin (**1a**) with suitable hydroxylamines to obtain the oximes **2a-c** [3]. The carboxylic acid group was then transformed into a hydroxamic acid by reaction with hydroxylamine hydrochloride (Fig 2).

**Fig 3:**
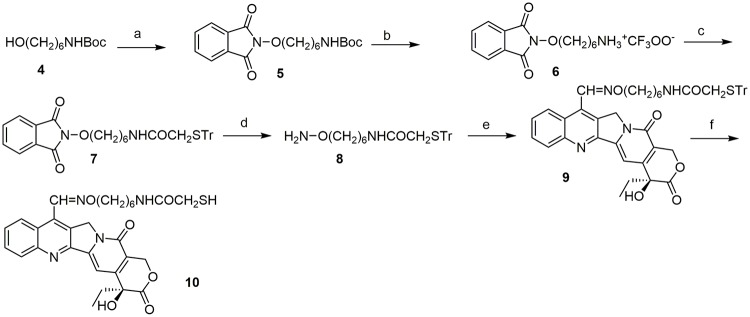
Synthesis of compound 10. Reagents and conditions: a) N-hydroxyphthalimide, PPh_3_, DIAD, THF, 63%; b) TFA, CH_2_Cl_2_, 94%; c) TrSCH_2_COOH, EDCI, DMAP, TEA, CH_2_Cl_2_, 53%; d) NH_2_NH_2_, CH_3_OH, 83%; e) 7-formylcamptothecin, EtOH, 91%; f) TFA, Et_3_SiH, CH_2_Cl_2_, 80%.

The CPT derivatives in clinical practice, Topotecan and Irinotecan, have a hydroxy group at C-10. In order to elucidate the contribution of this group, we prepared the 10-OH substituted analogue of compound **3b** (e.g. compound **3d**) starting from 7-formyl-10-hydroxy-CPT (**1b**) [27] (Fig 2). Furthermore, we decided to investigate the effect of the replacement of the hydroxamic acid moiety with a different HDAC Zn-binding group. Accordingly, we prepared compound **10**, carrying a mercaptoacetamide group in place of the hydroxamic acid [37]. The synthetic route is described in Fig 3. N-Boc protected 6-aminohexanol **4** was reacted with N-hydroxyphthalimide in Mitsunobu conditions to give compound **5**, which was deprotected and subsequently coupled with S-tritylmercaptoacetic acid [38]. The obtained hydroxylamine **8** was reacted with 7-formylcamptothecin [27] to give the oxime **9**, which after removal of trityl group by TFA, gave mercaptoacetamide **10**.

A series of similar compounds, having however the SAHA-like chain attached to the C-10 of CPT via an ether bond, has been recently reported by Oyelere and coworkers [39]. Another series of 10-O-ether derivatives have been described in a patent [40]. The most active of these compounds, CY-700, has been prepared by us for comparison (see below).

### Molecular modeling studies

#### DNA-Top1 complexes

**Fig 4:**
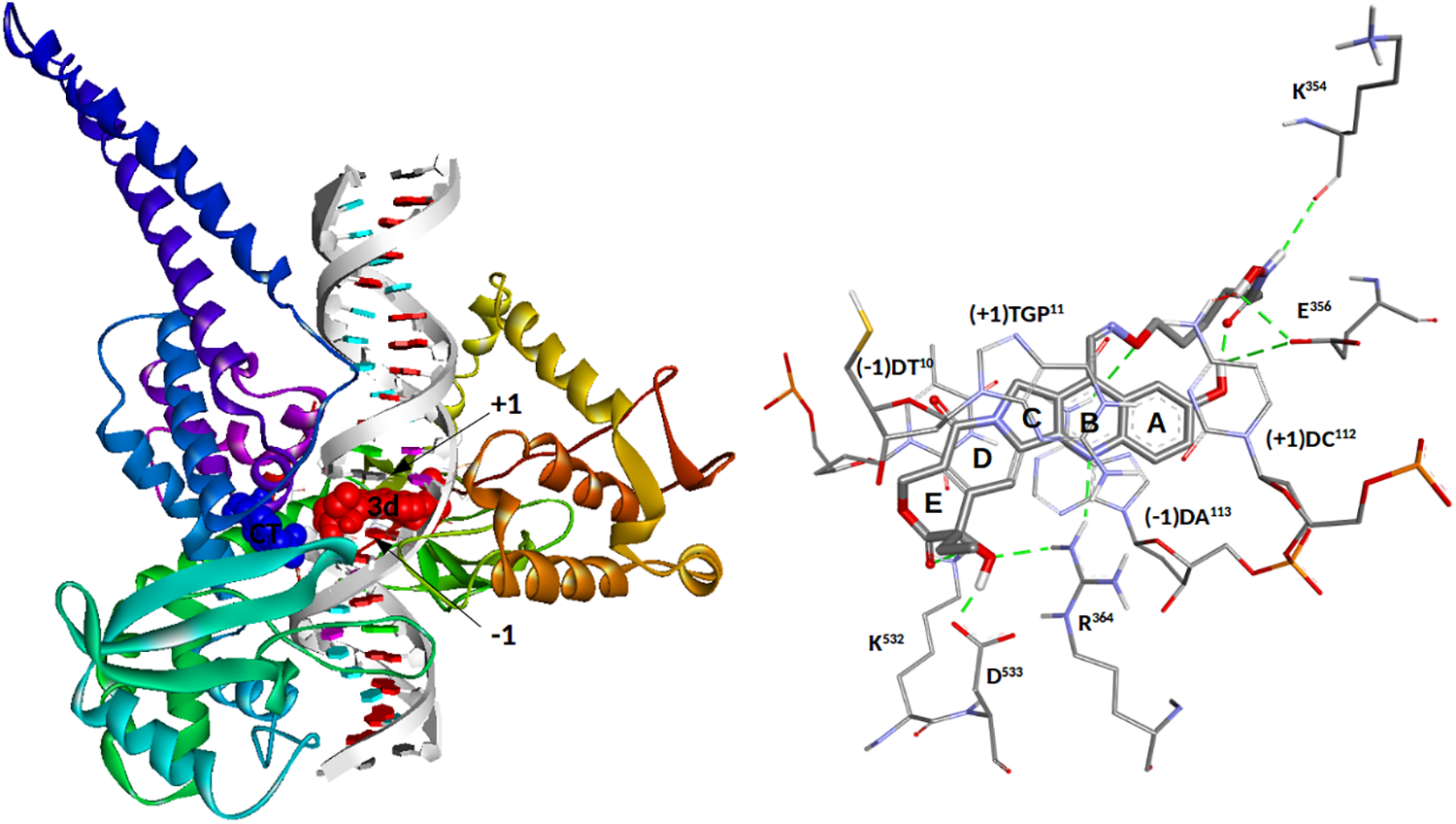
(Left)—Schematic representation of the proposed top-score binding mode for compound **3d** in the DNA-Topoisomerase-I complex. Topoisomerase-I is represented as Backbone Ribbon colored from blue (N-term) to red (C-term), while nucleic acids are represented as an arrow along the backbone pointing toward the C3'-end, and sugar groups and bases as boxes (A in red, C in violet, G in green and T in blue). The **3d** ligand and the catalytic tyrosine (CT) are rendered in CPK (**3d** in red and CT in blue). (Right)—Hypothetical binding mode for the Top-score conformation of **3d** in complex with DNA-Topoisomerase-I, with the aminoacids side chains relevant to the discussion rendered in line and the ligands in stick. Hydrogen bonds are shown as green dotted lines.

Molecular modelling studies were performed to explore the binding mode for compounds **3a-d** and **10** to the DNA-Top1 complex. The compounds are properly positioned inside the intercalation binding site, created by conformational changes of the phosphodiester bond between the +1 (upstream) and -1 (downstream) base pairs of the uncleaved strand, which effectively ‘‘open” the DNA duplex, with multiple strong π-π stacking interactions between the aromatic A, B and D rings of the camptothecin and both the -1 (T^10^-A^113^) and +1 (G^11^-C^112^) base pairs (Fig 4).

Compounds **3b** and **3d** show a hydrogen bond between the nitrogen atom N_1_ of ring B and R^364^NH_2_ residue, which also forms an additional hydrogen bond between R^364^NH_1_ and the OH group of the lactone ring E of all five compounds (see Table 1). The lactone ring is involved in other two interactions with the DNA-Top1 target. The first hydrogen bond, common to all five compounds, still involves the OH group of the lactone ring and D^533^O_δ2_. The other interaction is represented by a bidentate hydrogen bond formed between K^532^N_ζ_H_2/3_ and the C = O group of **3a**, **3c**, **3d** and **10**, while **3b** is able to form a single hydrogen bond with K^532^N_ζ_H_2_ only. The interaction pattern of the compound **3d** within the binding site is completed by a hydrogen bond between the camptothecin phenolic 10-hydroxy and E^356^O_ε2_, an interaction clearly impossible for the other compounds. Moreover, the phenolic 10-hydroxy of **3d** forms a strong intramolecular hydrogen bond with the carboxyl group of the side chain (1.66Å), increasing the stability of the complex which is the best among those studied in this paper.

**Table 1:** Binding free energies (kcal/mol) and selected distances (in Å) between the five ligands and the residues of the DNA-Top1 system.

**Interactions occouring within the binding site**											
	ΔG_binding_	K^532^N_ζ_H_2_	K^532^N_ζ_H_3_	D^533^O_δ2_	R^364^NH_1_	R^364^NH_2_	E^356^Oε_2_	(-1)T^10^	(-1)A^113^	(+1)G^11^	(+1)C^112^
**3a**	-9.21	2.66	2.68	2.32	2.43	-	-	π-π	π-π	π-π	π-π
**3b**	-10.47	-	2.37	2.05	2.61	2.83	-	π-π	π-π	π-π	π-π
**3c**	-9.24	2.70	2.82	2.44	2.43	-	-	π-π	π-π	π-π	π-π
**3d**	-11.82	2.70	2.61	1.64	2.30	2.67	2.95	π-π	π-π	π-π	π-π
**10**	-9.08	2.85	2.83	2.60	2.32	-	-	π-π	π-π	π-π	π-π
**Interactions involving the rear region of the binding site**											
	K^425^N_ζ_H_1_	K^425^N_ζ_H_2_	Y^426^O	E^356^Oε_2_	E^356^N	K^354^O	W^416^Nε_1_	(-1)A^113^N_7_	(-1)A^113^H_61_	(-1)A^113^H_62_	(-1)A^114^H_61_
**3a**	2.29	-	1.68	-	-	-	-	-	-	2.86	-
**3b**	2.82	1.75	-	-	-	-	-	2.18	2.93	2.92	1.89
**3c**	-	-	-	1.63	1.98	1.56	-	-	-	2.44	-
**3d**	-	-	-	2.55	-	2.57	-	-	-	2.88	-
**10**	-	-	-	-	-	-	1.94	-	-	2.68	-

Stacking interactions between the aromatic rings of camptothecin and the -1 (T^10^-A^113^) and +1 (G^11^-C^112^) base pairs are indicated with π-π.

The side chains of the five compounds are located in the rear region of the binding site, resting on the DNA duplex and forming a series of interactions that contribute to the stability of the complexes (Table 1). The only interaction common to all the studied compounds is the hydrogen bond between (-1)A^113^H_62_ and the oxygen of the oxime group, that only in **3b** forms a second hydrogen bond with (-1)A^113^H_61_. The C = O group of the **3a** side chain interact with K^425^N_ζ_H_1_, while **3b** forms a bidentate bond with both K^425^N_ζ_H_1_ and K^425^N_ζ_H_2_. The interaction with K^425^ is absent in **3c-d** and **10**, where the C = O group is rotated to form a hydrogen bond with W^416^N_ε1_.

The interaction of the hydroxyamic NH group with the target is present only for compounds **3a**, **3c** and **3d**, involving however different residues: **3a** with Y^426^O, **3c** with E^356^O_ε2_, **3d** with K^354^O. Finally, the interaction pattern of the side chains is completed by the interactions of the hydroxamic OH group with the DNA-topoisomerase-I system, present only in **3b**, **3c** and **3d**, but also in this case with different residues: **3b** with (-1)A^113^N_7_, (-1)A^114^H_61_ and K^425^N_ζ_H_2_, **3c** with E^356^N and K^354^O, **3d** with E^356^O_ε2_.

#### HDAC-II complexes

The complexes are obtained by molecular docking of **3a-d** and **10** using the Class I Rpd3-like protein HDAC2 as a model.

**Fig 5:**
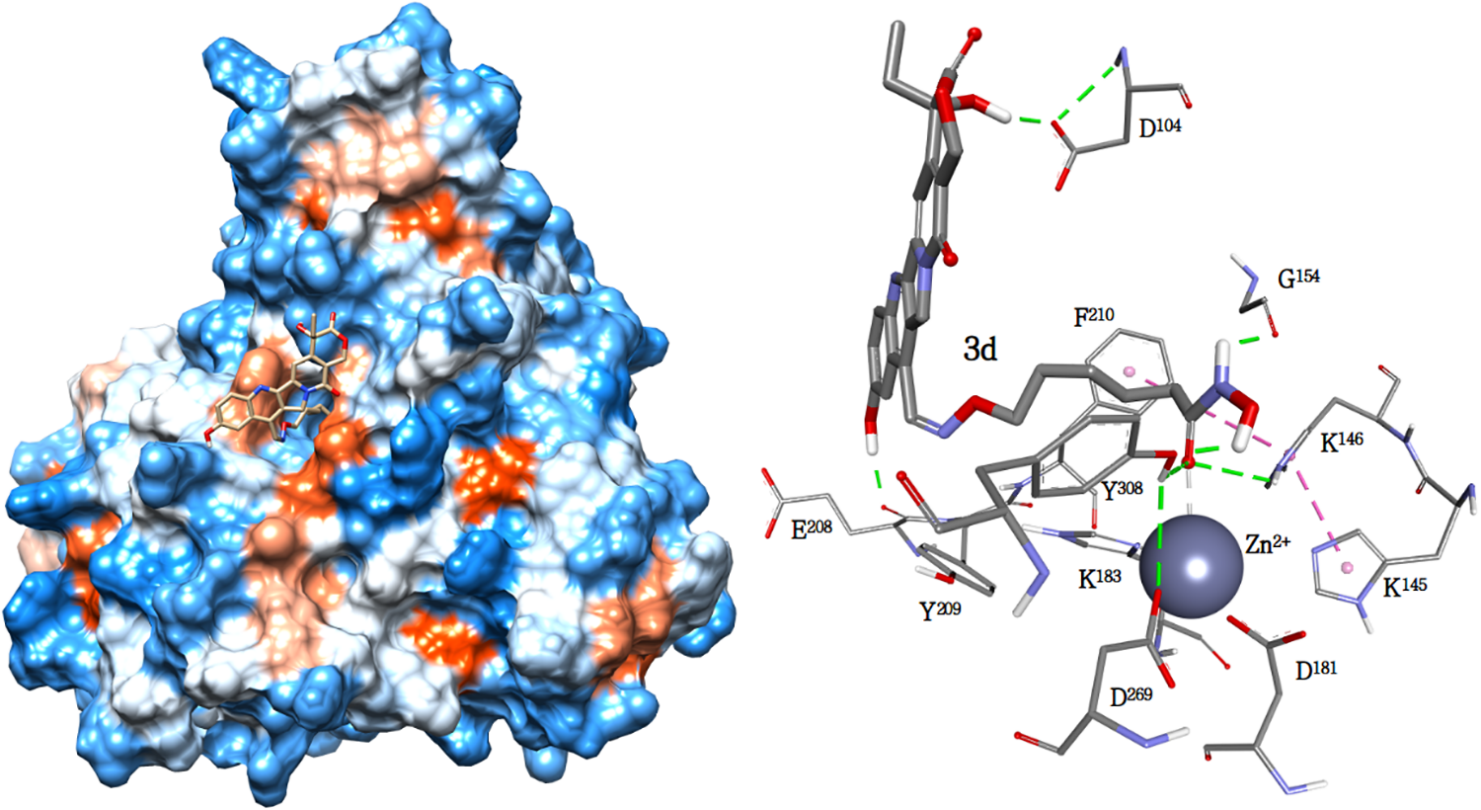
(Left)—Solvent Accessible Surface (SAS) representation of HDAC bond to **3d** (in stick). The portions of molecule interacting with the binding site is delimited by the oxime group, located near the entrance of the cavity. (Right)—Binding mode of the top-score conformation of **3d** to HDAC-II, with the aminoacids side chains relevant to the discussion rendered in line and the ligand in stick. Hydrogen bonds are shown as green dotted lines.

While the **3a-d** series shows minor structural modification in the linker length, **10** is rather different, due to the presence of the SH group, which might change the interactions with the catalytic zinc ion that is essential for enacting the enzyme. Apart from these differences, all the ligands enter the binding site in a very similar way, with the functionalized side chain inside the cavity and the camptothecin system that interacts with the external surface near the entrance to the cavity (Fig 5).

As expected, the docking results of **3a-d** were very similar and their side chains are highly superimposed. Their common interactions include a hydrogen bond from the hydroxamic NH towards G^154^O and two interaction involving the hydroxamic C = O group; a hydrogen bond with H^183^H_δ1_ and the chelation of the catalytic Zn^2+^ ion (Table 2). The residue Y^308^ is directly involved only in the binding of the compounds having the spacer with n = 5 (**3b** and **3d**), through a hydrogen bond involving Y^308^O and the C = O group of both compounds, and a further hydrogen bond between Y^308^O and the hydroxamic OH of the **3d** compound alone, which further stabilizes its conformation. Compound **10** still exhibits the interactions with G^154^ and H^146^, but there is no hydrogen bond with Y^308^. The loss of the interaction with Y^308^ is almost certainly attributable to the non-optimal spacer length, considering that the same effect is also found in compounds **3a** and **3c**. Furthermore, unlike **3a-d** compounds, in **10** the zinc ion is complexed by the sulfur atom, which also interacts with rings of H^145^, H^146^ and Y^308^, forming three Pi-Sulfur interactions.

**Table 2:** Binding free energies (kcal/mol) and distances (in Å) between the five ligands and HDAC II residues forming the catalytic binding site.

		Interactions occouring within the binding site				Interactions involving Camptothecin				
	ΔG_binding_	G^154^O	H^146^Hε_2_	Y^308^OH	Zn^2+^	D^104^O_δ1_	E^208^O	Y^209^N	Y^209^	F^210^
**3a**	-7.53	2.01	2.57	-	2.86	-	-	-	π-π	π-π
**3b**	-7.66	2.03	2.91	1.97	2.53	-	-	-	π-π	-
**3c**	-7.36	2.00	2.32	-	2.90	-	-	-	π-π	-
**3d**	-7.89	1.90	2.76	1.91 (OH) 2.14 (C = O)	2.74	1.56	1.98	-	-	-
**10**	-7.78	2.35	2.51	-	2.54	-	-	3.01	-	-

Stacking interactions are indicated with π-π.

The portions of **3a-d** and **10** interacting with the binding site are delimited by the oxime group, which in fact lies in all cases at the entrance of the cavity. The camptothecin chain leans to rest on the outer surface of the receptor to form interactions with residues at the rim of the pocket. For **3a-c** the only observed π-π stacking interactions are with the residue Y^209^ (**3a-c**), while only **3a** is able to form a pi-pi stacking with F^210^, evidently aided by the shorter linker that forces the camptothecin skeleton to approach the surface. In contrast, compound **10** does not show π-π stacking interactions with residues at the rim of the pocket, but is still anchored to the target surface by a hydrogen bond between the camptothecin carbonyl oxigen in 17 and Y^209^N.

In compound **3d**, the presence of the 10-hydroxy group and the optimal spacer length makes it once again unique among the studied compounds. These two structural features provide **3d** with the possibility of tightly interacting with the external surface through two strong hydrogen bonds, one between the OH of the lactone E ring and D^104^O_δ1_ and the other between the phenolic 10-hydroxy and E^208^O.

**Fig 6:**
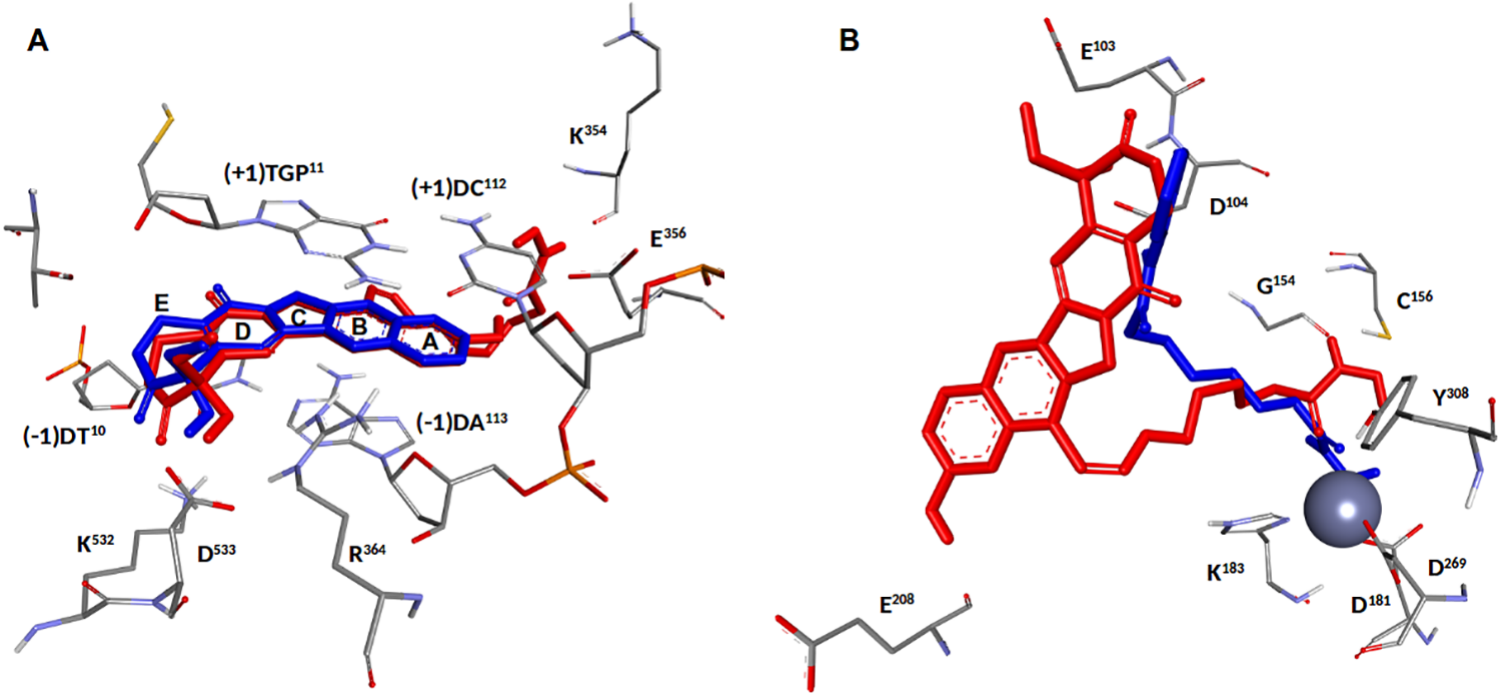
**(A**)–Superimposition of the **3d** (in red) and CPT (blue) structures in the catalytic site of Top1. (**B**)—Overlap of **3d** (red) and SAHA (blue) within the HDAC-II catalytic site.

Finally, compound **3d** was superimposed to the reference ligands CPT and SAHA in the catalytic sites of Top1 and HDAC II, respectively (Fig 6A and 6B). Fig 6A shows the superimposition of **3d** and CPT in the catalytic site of Top1. The two conformations are almost perfectly superimposed, with the exception of the D-E rings, which in **3d** move close to K^532^ and D^533^ to obtain a better interaction. The overlap of **3d** and SAHA within the HDAC-II catalytic site is reported in Fig 6B. Although the functional groups are different, the two molecules behave similarly within the cavity, both interacting with the same residues and chelating the Zn^++^ ion. The two structures diverge as one moves away from the centre of the cavity, as a result of the different interaction with the surface of the enzyme. This behaviour can be explained considering the different dimensions and properties of the **3d** and SAHA terminal groups.

Overall, the results obtained showed that hybrids with a 5/6 carbon chain are capable of forming stable complexes with both HDAC and Top1. Compound **3d** has the most favourable interactions and its promising binding mode provides a computational validation to our plan to combine the CPT and SAHA-like motifs in a single molecule.

### Biological activity evaluation

The antiproliferative activity was evaluated on a series of human tumor cell lines (NCI-H460: non small cell lung cancer; CAPAN 1: human pancreatic ductal adenocarcinoma; A431: human epidermoid carcinoma; HeLa: human epithelioid cervix carcinoma; HT29: human colon adenocarcinoma; DU145: human prostate carcinoma; HepG2: human liver hepatocellular carcinoma; A2780: human ovarian carcinoma; A2780-Dox: human doxorubicin-resistant ovarian carcinoma). The activity was assessed after 72 h with the SRB assay.

Compounds **3a-c**, having different linker length, showed significant antiproliferative activity, suggesting that the chain was flexible enough to effectively position the molecule within the enzymes’ active sites. Compound **3d**, the 10-hydroxy substituted derivative of **3b**, showed a very potent antiproliferative activity as well. In fact, it displayed a cytotoxic activity in the nanomolar range (0.03 μM < IC_50_ < 0.4 μM), while SAHA (IC_50_ > 0.9 μM), Irinotecan (IC_50_ > 1.5 μM), and the reference compound dual inhibitor CY-700 (0.3 μM < IC_50_ < 3.3 μM) were all less effective (Table 3). Compound **10**, carrying a Zn-binding group different from the hydroxamic acid, maintained a significant antiproliferative activity.

**Table 3:** Human tumor cell lines exposed to Top1, HDAC, and dual Top1/HDAC inhibitors.

	NCI-H460	Capan 1	A431	HeLa	HT29	DU145	HepG2	A2780	A2780-Dx	RI
Cpd^a^	IC_50_ ± SD (μM)									
**Irinotecan**	2.4 ± 0.1	1.5 ± 0.1	4.1 ± 0.1	5.8± 0.6	4.8 ± 0.4	2.8 ± 0.07	4.0 ± 0.8	3.8 ± 0.5	20.1± 2.9	5.3
**SAHA**	5.0 ± 0.2	1.8 ± 0.2	3.5 ± 0.1	2.7 ± 0.2	2.2 ± 0.3	0.87 ± 0.03	1.54 ± 0.1	2.1 ± 0.2	3.3 ± 0.6	1.6
**CY700**	0.36 ± 0.02	0.96±0.08	0.79± 0.1	3.3± 0.9	1.2 ± 0.2	0.87±0.04	1.5 ± 0.4	2.5 ± 0.3	5.9 ± 1.0	2.4
**3a**	0.36 ± 0.10	0.05± 0.006	n.e.	n.e.	n.e.	n.e.	n.e.	n.e.	n.e.	
**3b**	0.023±0.004	0.006±0.0007	0.004±0.001	0.16±0.04	n.e.	n.e.	n.e.	0.008±0.002	0.27±0.070	31.7
**3c**	0.198±0.18	0.043± 0.003	0.026±0.001	2.4 ± 0.5	0.045±0.01	0.029±0.001	0.68±0.2	0.038±0.008	0.065±0.01	1.7
**3d**	0.27 ± 0.02	0.035 ± 0.002	0.14 ± 0.02	0.18±0.05	0.39±0.1	0.07±0.003	0.11±0.03	0.27 ± 0.08	1.8 ± 0.4	6.6
**10**	0.1 ± 0.01	n.e.	n.e.	n.e.	n.e.	n.e.	n.e.	0.21 ± 0.05	0.35 ± 0.1	1.7

^a^Human tumor cell lines were treated with various Top1, HDAC, and dual Top1/HDAC inhibitors and the anti-proliferative activity assessed after 72 h with the SRB assay. RI = Resistance Index. Ne = not evaluated.

Compounds **3b** and **3d** were selected for a deeper characterization of their target modulation and specificity.

Initially, a comparative study on different purified HDAC isoforms was carried out (Table 4).

**Table 4:** Inhibition of HDAC isoforms by compounds 3b, 3d.

Compound^a^	HDAC1	HDAC2	HDAC4	HDAC6	HDAC10
**3d**	5,26E-08	9,33E-08	n.a	1,56E-09	2,24E-07
**3b**	4,30E-08	7,62E-08	n.a.	3,87E-09	9,99E-08
**Trichostatin A**	1,244E-08	2,36E-08	-	2,09E-09	3,20E-08
**TMP269**	--	--	3.53E-07	--	--

^a^IC_50_ values were calculated using the GraphPad Prism 4 program based on a sigmoidal dose-response equation.

n.a. = not active. Trichostatin A and TMP269 were used as reference standards

Both compounds showed a significant activity against four purified HDAC isoforms, representative of class I (HDAC 1, 2) and Class IIb (HDAC 6, 10) HDACs, with IC_50_ comparable to those of the reference compound Trichostatin A, without substantial selectivity. Conversely, both compounds were inactive against HDAC 4 (representative of Class IIa HDACs) (Table 4). This result was not unexpected, as it is well known that SAHA inhibits class IIa enzymes at concentrations that are not clinically significant [41].

**Fig 7:**
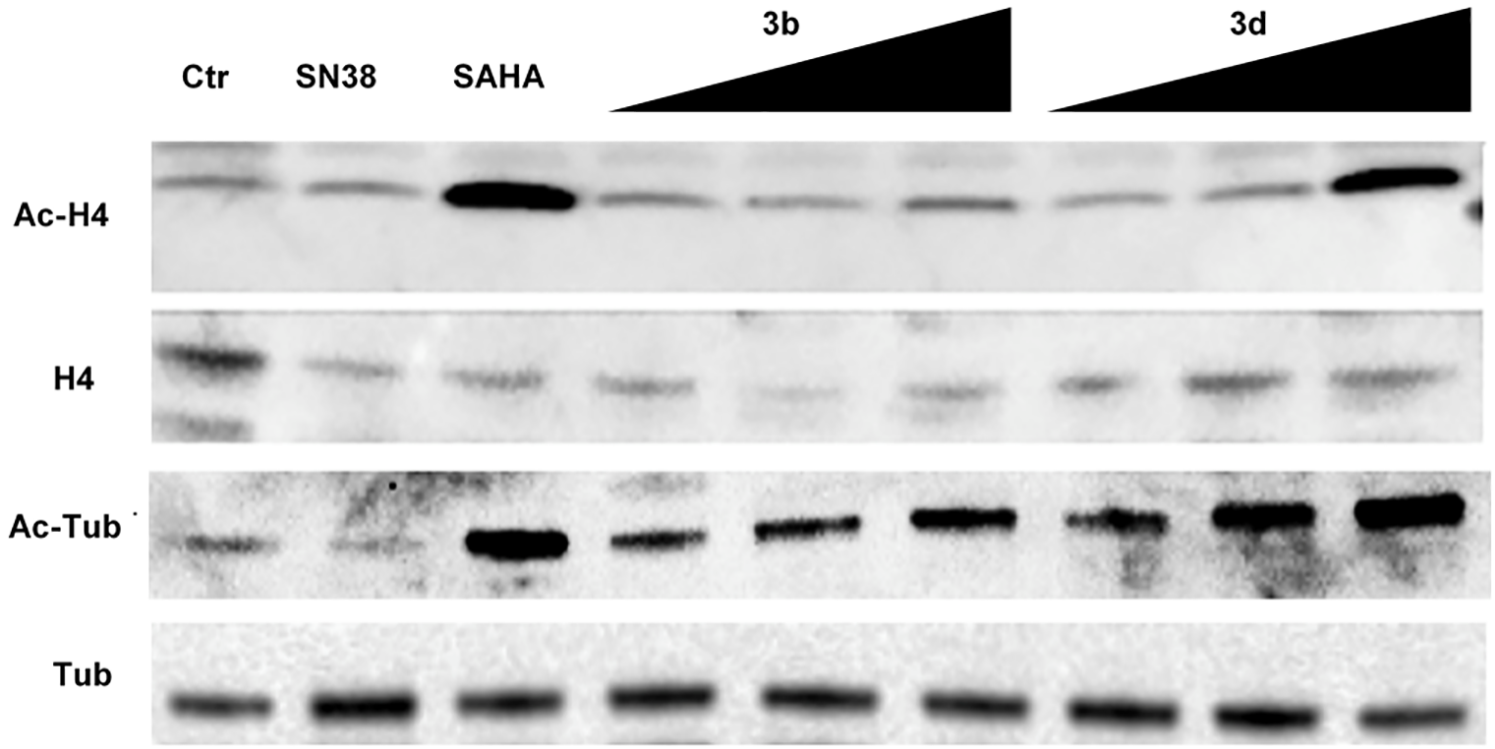
Effects of compounds 3b, 3d, SN38 and SAHA (reference compounds) on acetylation of tubulin and histone H4 in NCI-H460 cells. Cells were treated for 24 h with SN38 (0.05 μM), SAHA (5 μM), **3b** and **3d** (0.125–0.2–0.5 μM). Cells were lysed in 0.5% NP-40 and 40 μg of total proteins were loaded on NuPAGE 4–12% (Invitrogen), transferred to a nitrocellulose paper and probed with specific antibodies. Acetylated Histone H4 (Ac-H4); Histone H4; Acetylated Tubulin (Ac-H4); Tubulin.

Subsequent analyses were conducted to verify whether **3b** and **3d** effectively targeted HDACs and Top1. To explore the HDAC inhibitory activity of the selected compounds, NCI-H460 cells were exposed to **3b** and **3d** (at different concentrations: 0.125, 0.2, 0.5 μM) and to the reference compounds SN38 (the active metabolite of Irinotecan) and SAHA (0.05 and 5 μM, respectively, corresponding approximately to their own IC_80_). The accumulation of both acetylated histone H4 and acetylated tubulin was investigated (Fig 7). Compound **3d**, similarly to SAHA, showed the characteristics of a Pan-HDAC inhibitor, as revealed by H4 and tubulin acetylation in a dose dependent manner. Compound **3b**, at a concentration corresponding to its IC_80_ (0.5 μM), showed to be less potent than **3d** on both targets, particularly on H4 (Fig 7).

**Fig 8:**
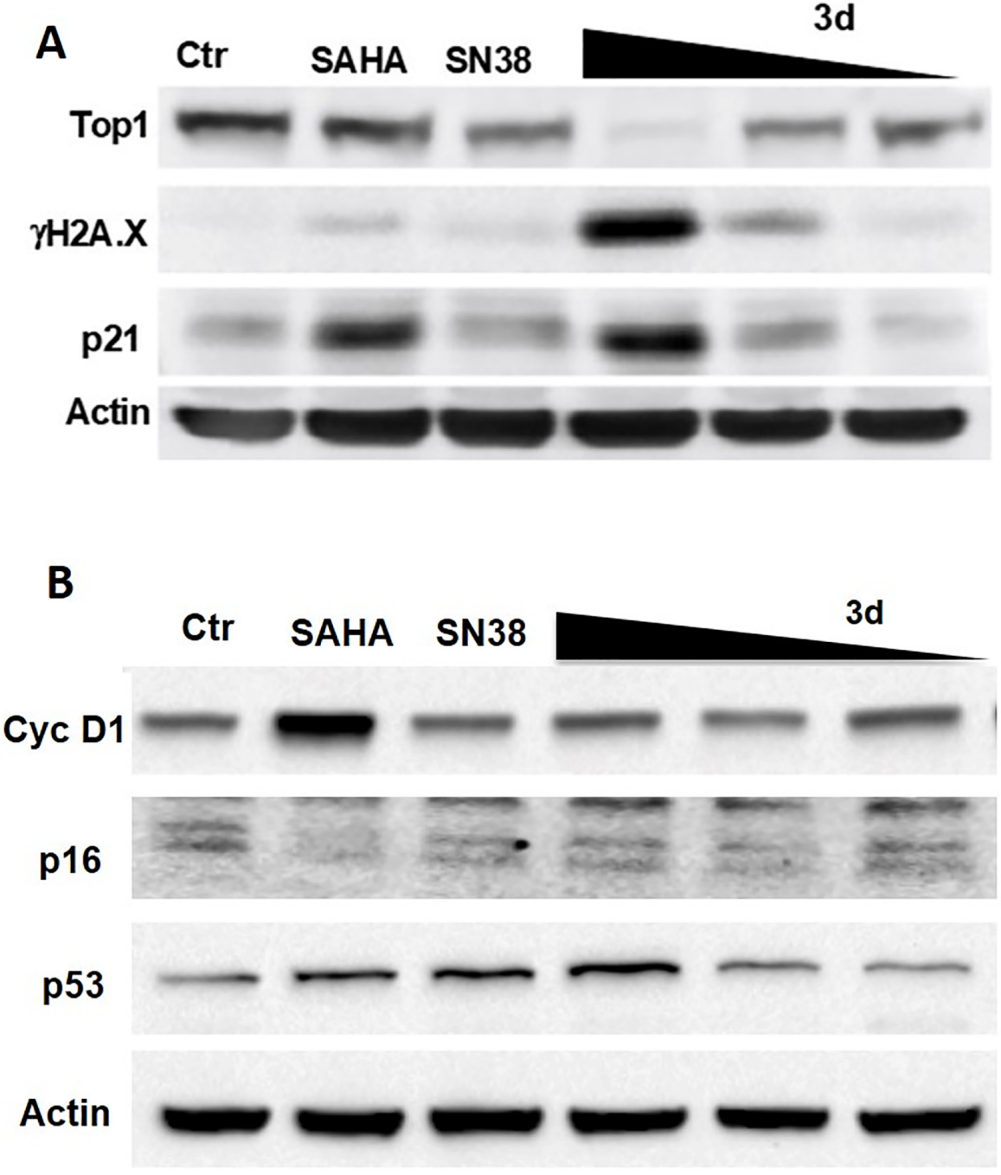
Effects of compound 3d, SN38 and SAHA (reference compounds) on Top1, H2A.X, p21 (A), CycD1, p16, p53 (B) in NCI-H460 cells. H460 cells were treated for 24 h with SAHA (5 μM), SN38 (0.05 μM), and **3d** (0.125–0.2–0.5μM). Cells were lysed in 0.5% NP-40 and 40 μg of total proteins were loaded on NuPAGE 4–12% (Invitrogen), transferred to a nitrocellulose paper and probed with specific antibodies.

Following on from these results, **3d** was further investigated to assess its ability to inhibit Top1. Interestingly, the compound drastically reduced the expression of Top1 (comparable to SN38) (Fig 8A). This finding is consistent with recent literature, where there is evidence that treatment with topoisomerase inhibitors significantly decreases the Top I expression [42]. In addition, **3d** induced p53 activation (Fig 8B) and DNA damage at a dose corresponding to its IC_80_, confirmed by H2A.X induction (Fig 8A). Interestingly, SN38 and SAHA at equivalent doses appeared less effective in p53 up-regulation and completely ineffective in H2A.X induction (Fig 8A and 8B), thus suggesting that the **3d** dual targeting activity cooperates in the DNA damage induction.

Moreover, P16 and CycD1 were down and up-regulated, respectively, by SAHA, while **3d** and SN38 didn’t show any effect. Interestingly, SAHA-induced block of G2/M progression observed in our experiment (see below) and its induction of p21, p53 and Cyclin D1 suggest an effect on senescence pathways activation, as recently reported [43].

Furthermore, similarly to SAHA, **3d** activated the expression of p21, probably as a consequence of its HDAC inhibitory activity (Fig 8). Taken together, these results suggest that **3d** possesses a dual-inhibitor characteristic, acting at cellular level both as HDAC and Top1 inhibitor.

To assess the antitumor potential of compound **3d**, a large screening on hematologic cancer cell lines was carried out (Tables 5 and 6). The compound showed a cytotoxic potency mostly in the nanomolar range (0.006 μM < IC_50_ < 4 μM), superior to SAHA (0.6 μM < IC_50_ < 9 μM), and Irinotecan (IC_50_ > 10 μM) in most cell lines. Interestingly, **3d** showed an activity superior to that of CY-700 (0.3 μM < IC_50_ < 10 μM), the most active compound described by Chen [40], taken as a reference standard of dual CPT-HDAC inhibitors. These data confirm the correctness of the choice of linking the HDAC-active chain to the position 7 of camptothecin.

**Table 5:** Human lymphoma and leukemia cell lines exposed to 3d and Top1, HDAC, and dual Top1/HDAC inhibitor CY700.

Cpd	MINO	MAVER-2	JECO-1	U-2932	OCI-LY3	L-428	KM-H2	DG-75	NB4
	IC_50_ ± SD (μM)								
**3d**	0.031±0.01	0.012±0.001	0.023±0.006	0.044±0.01	0.018±0.005	0.43±0.07	0.39±0.1	1.9±0.2	n.e.
Irinotecan	>10(36%)	0.74 ± 0.1	>10 (48%)	4.4 ± 0.8	> 10 (0%)	>10(0%)	>10(12%)	>10(0%)	1.0±0.3
CY700	0.37± 0.1	0.4 ± 0.08	1.0 ± 0.4	0.9 ± 0.3	0.34±0.04	1.7 ± 0.2	1.5 ± 0.5	>10(18%)	0.24±0.06
SAHA	1.1 ± 0.3	5.4 ± 1.2	2.6 ± 0.7	1.1 ± 0.2	0.99 ± 0.3	2.3± 0.5	1.2± 0.2	2.1 ± 0.2	3.3± 0.6

Human hematologic cancer cells were exposed to various Top1, HDAC, and dual Top1/HDAC inhibitors and the anti-proliferative activity assessed after 72 h with the MTT assay. Ne = not evaluated.

**Table 6:** Human myeloma, leukemia and lymphoma cell lines exposed to 3d and SAHA.

Cell line	SAHA	3d
IC_50_ ± SD (μM)		
K 562	1.53 ± 0.4	0.94 ± 0.2
ARH-77	3.9 ± 0.5	0.21 ± 0.06
THP1	3.9 ± 1.4	0.22 ± 0.09
MV4-11	3.84 ± 0.8	2.73 ± 0.3
Jurkat-cp	1.99 ± 0.2	0.23 ± 0.05
U937	1.62 ± 0.3	0.18 ± 0.05
RAJI	1.73 ± 0.6	0.077 ± 0.02
RAMOS	3.9 ± 0.7	0.99 ± 0.1
REC-1	0.94 ± 0.2	0.18 ± 0.04
Z-138	0.7 ± 0.1	0.006 ± 0.001
RPMI-8226	1.98 ± 0.5	4.1 ± 1.4
H929	0.56 ± 0.07	1.1 ± 0.4

Human hematologic cancer cells were exposed to 3d and SAHA and the anti-proliferative activity assessed upon 72h with the MTT assay.

Lastly, we tested **3d** against a series of primary malignant pleural mesothelioma (MPM) cell lines (from our cell bank collection).

We compared **3d** to drugs in clinical use for the treatment of MPM, such as Cisplatin, and drugs such as Irinotecan, SAHA, Gefitinib, 5-FU, which failed to increase the survival of MPM patients when investigated in clinical trials [44].

Gratifyingly, **3d** showed a potent antiproliferative effect, with IC_50_ ranging from 0.05 to 11 μM, which was much higher than SAHA (4 μM < IC_50_ < 12 μM) and Irinotecan (IC_50_ > 7 μM). Moreover, **3d** resulted more effective than Gefitinib (14 μM < IC_50_ < 38 μM), cisplatin (1.2 μM < IC_50_ < 8.0 μM), and 5-FU (18 μM < IC_50_ < 200 μM), as well (Table 7). These results are remarkable, considering that mesothelioma still remains an incurable cancer, and new therapeutic approaches against the disease are urgently needed [45].

**Table 7:** Human primary mesothelioma cell lines exposed to 3d and reference antitumor drugs.

Cpd	EPITHELIOID					BIPHASIC		SARCOMATOID	
	IC_50_ ± SD (μM)								
	MM288	MM317	MM404	MM473	MM481	MM487	MM491	MM432	MM472
**3d**	0.48±0.1	0.85±0.2	1.4 ±0.3	2.1±0.5	11.7±1.2	0.2±0.05	0.87±0.3	0.16±0.03	0.048±0.01
**Irinotecan**	14.9±2.0	7.0 ± 1.0				9.4 ± 0.7		10.0±2.0	
**SAHA**	3.8 ±0.2	11.2±0.9	8.8± 1.2	8.5± 0.4	11.5±0.4	7.4 ± 0.4	10.7±0.4	6.9 ± 1.0	4.6 ± 0.8
**Gefitinib**	38.7±1.4	26.1±2.9	28.4±2.4	18.4±0.7	31.2±1.0	15.0±0.5	14.0±0.7	17.1 ± 1.0	19.1 ± 0.5
**Cisplatin**	8.1 ± 0.3	4.2 ± 0.6	2.4 ± 0.5	2.4 ± 0.2	7.0 ± 1.5	3.0 ± 0.5	6.5 ± 0.9	5.0 ± 0.8	1.26 ± 0.1
**5-FU**	>200(35%)	17.9±3.1	52.2±8.7	35.2±8.4	27.4±2.2	>200(48%)	44.0±10.3	>200(34%)	40. ± 10.5

Human primary mesothelioma cells were exposed to **3d** and different anti-tumor compounds. Anti-proliferative activity was assessed upon 72 h with the SBR assay.

Compound **3d** was also tested, comparing it with Irinotecan and SAHA, on NCI-H460 NSCLC cell line to assess the effect on cell cycle progression and induction of apoptosis. FACS analysis revealed a comparable effect of **3d** and Irinotecan on cell cycle distribution, with a steady block of treated-cells in the S phase, while SAHA preferably arrested cells in G2/M. Interestingly, **3d** also had cytotoxic activity, with induction of apoptosis (sub-G_0/1_ population) that was lacking in cells treated with Irinotecan and SAHA (Table 8).

**Table 8:** Flow cytometric evaluation of human NCI- H460 NSCLC cells exposed to 3d and reference antitumor drugs.

NCI-H460^a^	G_0/1_ (%)	S (%)	G_2_/M (%)	Sub-G_0/1_ (%)
Ctr	71.0	23.0	5.9	2.4
**3d**	12.1	76.0	11.9	15.2
Irinotecan	7.9	89.0	3.2	2.7
SAHA	65.2	11.7	23.1	1.8

^a^Tumor cells were exposed for 72 h to **3d** and reference anti-tumor drugs (at their own IC_80_ dose), stained with PI, and processed on a FACScan flow cytometer. The CellQuest software was used to acquire data and assess the sub-G_0/1_ population, while cell cycle analysis was performed with the ModFit software.

The efficacy of the new dual inhibitor **3d** was evaluated on human mesothelioma primary cell line MM473-Luc orthotopically xenografted in CD-1 nude mice. This cell line was stably transfected with luciferase and was suited for the observation of tumor growth by chemoluminescence analysis. After tumor cells injection and with tumors in the growth phase (day 15), mice were intravenously treated with **3d** (45 mg/kg, q4dx3w), Irinotecan (35 mg/kg, q4dx3) or vehicle as negative control (treatments ended at day 32). The tumor volume was measured as BLI signal and expressed as average radiance.

**Fig 9:**
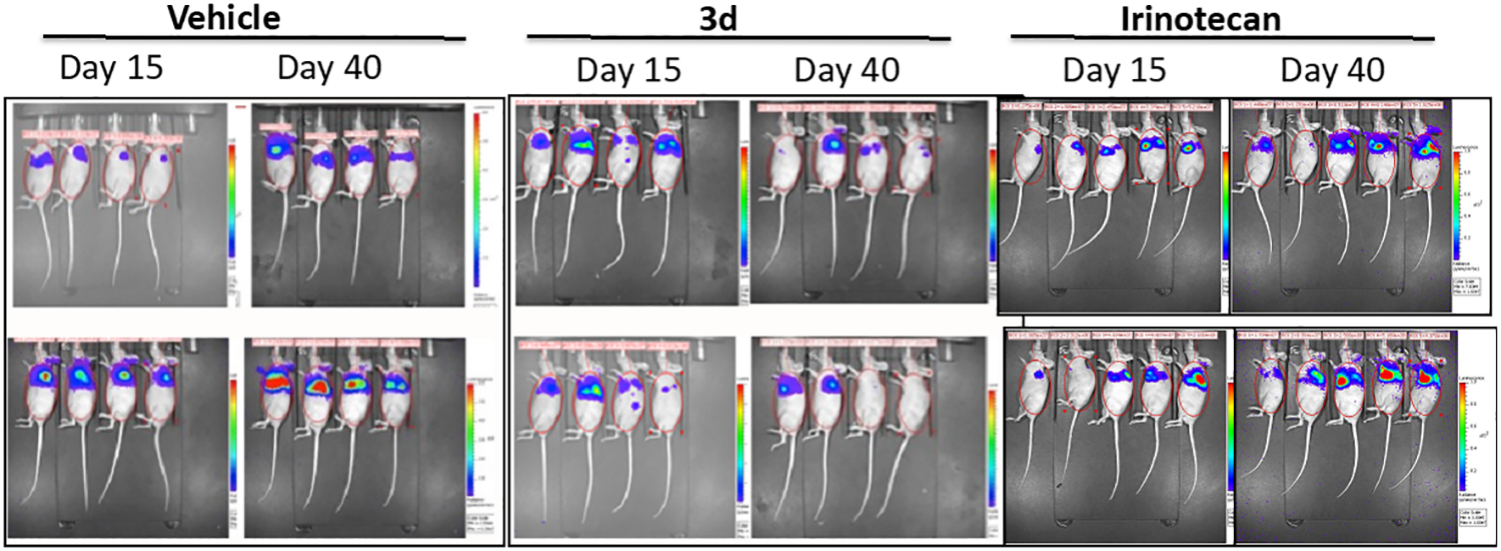
Efficacy of 3d in an orthotopic xenograft model of human epithelioid mesothelioma. Bioluminescence signal from mice injected with MM473 expressing luciferase at day 15 (first treatments) and day 40 (8 days after the last treatment).

**Fig 10:**
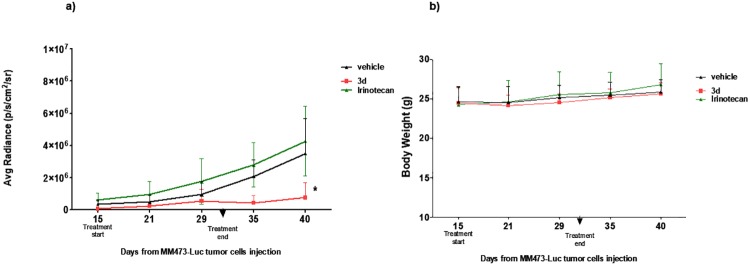
Efficacy of 3d in an orthotopic xenograft model of human epithelioid mesothelioma. a) bioluminescence signal of experimental groups throughout the experimental period; b) body weight curve. Statistical analysis performed using non parametric Mann-Whitney test (U) **3d** groups versus Vehicle *p<0,05.

The molecule administered had a strong antitumor activity as shown in Figs 9 and 10. Moreover, the tumor chemiluminescence in animals treated with **3d** indicated that the compound acts by both reducing the tumor growth progression and, in some cases, by reducing the tumor size until the disappearance of the bioluminescent signal (8 days after the end of treatments) (Figs 9 and 10). Conversely, Irinotecan had only a marginal antitumor effect (not significant, p>0.5) when administered at 35 mg/kg. Interestingly, the antitumor activity was observed at doses that appear well tolerated (no reduction in body weight was observed, data not shown).

## Conclusion

Synergistic effects have been reported on the use of HDAC and topoisomerase I inhibitors as a co-treatment on various cancer cells. In this study, new hydroxamic acid derivatives of camptothecin were evaluated as dual-action CPT-HDAC inhibitors. In particular, compound **3d** displayed a broad spectrum of antiproliferative activity, with IC_50_ values in the nanomolar range, on a series of human solid tumor, hematologic, and mesothelioma cell lines. The new hybrid molecule showed a potency superior to both HDAC inhibitor SAHA and Top1 inhibitor Irinotecan, increased antitumor activity and very high tolerability in *in vivo* human tumor models with respect to SAHA and Topotecan.

In molecular modelling studies, compound **3d** showed the most favourable interactions and its promising binding mode provided structural support to the molecular design and development of analogues. Overall, the docking interaction profiles of synthesized compounds were in accordance with the biological results, especially for selected compound **3d**, which was found to have significantly better interactions with both target proteins. This finding can be explained considering the simultaneous presence in **3d** of the phenolic -OH on ring A of camptothecin (10-hydroxy) and the right length of the linker (n = 5). These two structural features represent the driving force that makes **3d** a better ligand for both the DNA-Top1 binary complex and the HDAC-II active site.

The single dual-function molecule not only attacks the cancer cells from two distinct pathways simultaneously but also improves drug efficacy of the conventional CPT without increasing its dose-limiting toxicity. Therefore, it can be used for treating tumors susceptible to camptothecin drugs and/or to HDAC inhibitors, showing at the same time a good tolerability and low toxicity.
